# Supplementary material for: The Effects of Daily Temperature on Crime Events in Urban Hanoi, Vietnam Using Seven Years of Data (2013–2019)
Source: Int J Environ Res Public Health. 2022 Oct 26;19(21):13906. doi: 10.3390/ijerph192113906 (PMC9657037; doi:10.3390/ijerph192113906)
Supplement: Supplementary file 1 [file ijerph-19-13906-s001.zip › ijerph-1914676-supplementary.pdf]

Supplementary Material:

Linear and non-linear effects of Daily temperature and crime events in urban Hanoi, Vietnam using seven years of data (2013-2019)

Vu Thuy Huong Le [1,2], Jesse D. Berman [1], Quynh Anh Tran [2], Elizabeth V. Wattenberg [1], and Bruce H. Alexander [1,] *

[1] University of Minnesota School of Public Health, Division of Environmental Health Sciences, Minneapolis, Minnesota, USA
[2] Hanoi Medical University School of Public Health, Department of Environmental Health, Hanoi, Vietnam

Corresponding author
Bruce H. Alexander
University of Minnesota School of Public Health
balex@umn.edu

The statical models for estimate the linear effects of temperature on crime

The following quasi-Poisson statistical model was used to estimate the risk of a crime event for each 5 °C in daily temperature. The model was fitted separately for temperature measurements, each crime types and total crime

Model 0:

$$Log\ E(Y_t) = \delta + \varepsilon(Temp) + \phi(Season) + \gamma(Year)$$

Model 1:

$$Log\ E(Y_t) = \delta + \varepsilon(Temp) + \phi(Season) + \gamma(Year) + æ(Day) + \theta(Holiday)$$

Model 2:

$$Log\ E(Y_t) = \delta + \varepsilon(Temp) + \phi(Season) + \gamma(Year) + æ(Day) + \theta(Holiday) + þ(RH)$$

Model 3:

$$Log\ E(Y_t) = \delta + \varepsilon(Temp) + \phi(Season) + \gamma(Year) + æ(Day) + \theta(Holiday) + ÿ(PM25)$$

Model 4:

$$Log\ E(Y_t) = \delta + \varepsilon(Temp) + \phi(Season) + \gamma(Year) + æ(Day) + \theta(Holiday) + þ(RH) + ÿ(PM25)$$

where Y_t is the number of crimes on day t, δ is the intercept, ε is the regression coefficient for Temperature, and *Temp* is the temperature measurements. For long-term and seasonal trends, we controlled for season and year, where ϕ is the vector of coefficients for season, *Season* is the season indicator variable on day t, γ is the regression coefficient for year, and *Year* is the year indicator.

We will adjust for daily characteristics, such as the day of the week and holiday, where *æ* is the vector of coefficients for day of week, *Day* is the day of the week indicator variable on day t, θ is the regression coefficient for holiday status on day t, and *Holiday* is the binary variable which 1 is a public holiday.

Finally, we controlled for relative humidity and particulate matters, where *þ* is the vector of coefficients for relative humidity, *RH* is the relative humidity indicator variable on day t, *ÿ* is the regression coefficient for particulate matters, and *PM25* is the particulate matters indicator on day t.

The statical models for estimate the non-linear effects of temperature on crime

Distributed lag non-linear models (DLNM) were fit to estimate the relative risk of each 1 °C increase in daily temperature and each type of crime. The model was fitted separately for each crime types and total crime

$$Log\ E(Y_t) = \delta + ns(Tempt_{0\text{-}7}, 4) + \phi(Season) + \gamma(Year) + æ(Day) + \theta(Holiday)$$

where Y_t is the number of crimes on day t, δ is the intercept, + $ns(Tempt_{0\text{-}7},4)$ describe a natural cubic spline with 4 degrees of freedom and 7 lag days for temperature.

For long-term and seasonal trends, we controlled for season and year, where ϕ is the vector of coefficients for season, *Season* is the season indicator variable on day t, γ is the regression coefficient for year, and *Year* is the year indicator.

We will adjust for daily characteristics, such as the day of the week and holiday, where *æ* is the vector of coefficients for day of week, *Day* is the day of the week indicator variable on day t, θ is the regression coefficient for holiday status on day t, and *Holiday* is the binary variable which 1 is a public holiday.

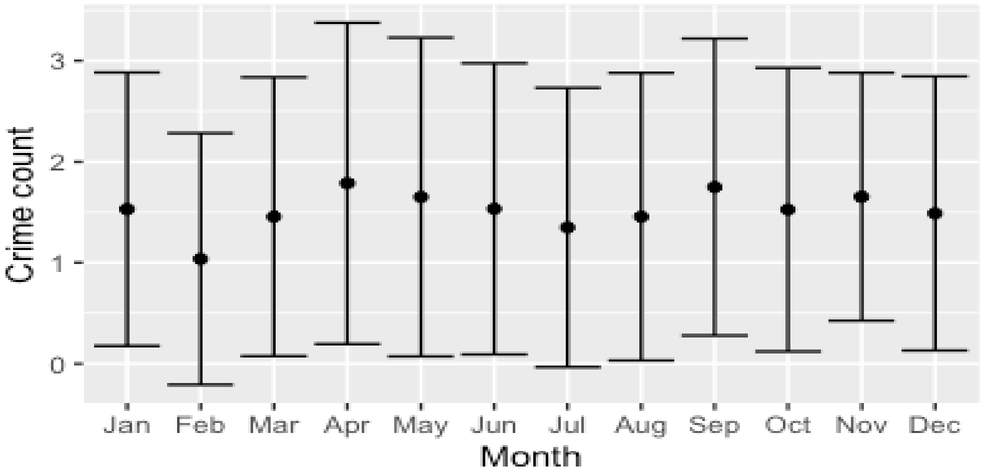

Figure S1: Monthly mean and standard deviations of crime count in urban Hanoi, 2013 – 2019

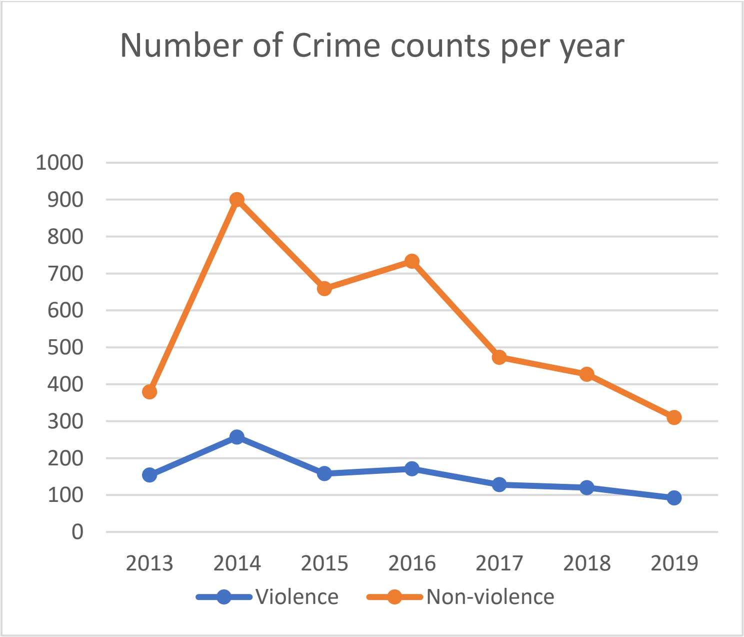

Figure S2: Yearly crime counts in urban Hanoi, Vietnam, from 2013-2019

Table S1: Daily temperature (degree Celcius) in 11 air monitors in urban Hanoi, Vietnam

Districts	**Mean**	**SD**	**Min**	**Median**	**Max**
Long Bien	25.05	5.44	6.97	26.13	37.31
Minh Khai	25.00	5.18	6.7	26.04	36.51
Trung Yen	25.18	5.19	7.99	26.22	35.67
Hoan Kiem	27.54	5.61	10.27	28.59	39.84
Kim Lien	27.70	5.69	10.35	28.77	40

My Dinh	28.06	5.76	10.59	29.25	39.85
Pham V Dong	27.85	5.56	10.64	28.92	39.52
Tan Mai	27.77	5.74	8.65	28.91	39.90
Tay Mo	27.56	5.66	7.86	28.62	38.99
Thanh Cong	27.47	5.60	9.08	28.46	39.88
Hang Dau	28.13	5.74	6.93	29.30	39.86

Table S2: The pair-wise correlation of daily mean temperature between each air monitor in 11 air monitors in urban Hanoi, Vietnam

Long Bien	1										
Minh Khai	0.88	1									
Trung Yen	0.90	0.87	1								
Hang Dau	0.90	0.85	0.89	1							
Hoan Kiem	0.95	0.89	0.90	0.95	1						
Kim Lien	0.88	0.88	0.91	0.96	0.99	1					
My Dinh	0.92	0.83	0.85	0.90	0.93	0.94	1				
Thanh Cong	0.93	0.87	0.89	0.95	0.97	0.98	0.92	1			
Tay Mo	0.93	0.88	0.89	0.95	0.98	0.99	0.93	0.97	1		
Tan Mai	0.93	0.88	0.90	0.95	0.98	0.99	0.93	0.98	0.99	1	
Pham V Dong	0.93	0.88	0.89	0.94	0.97	0.97	0.93	0.96	0.97	0.96	1

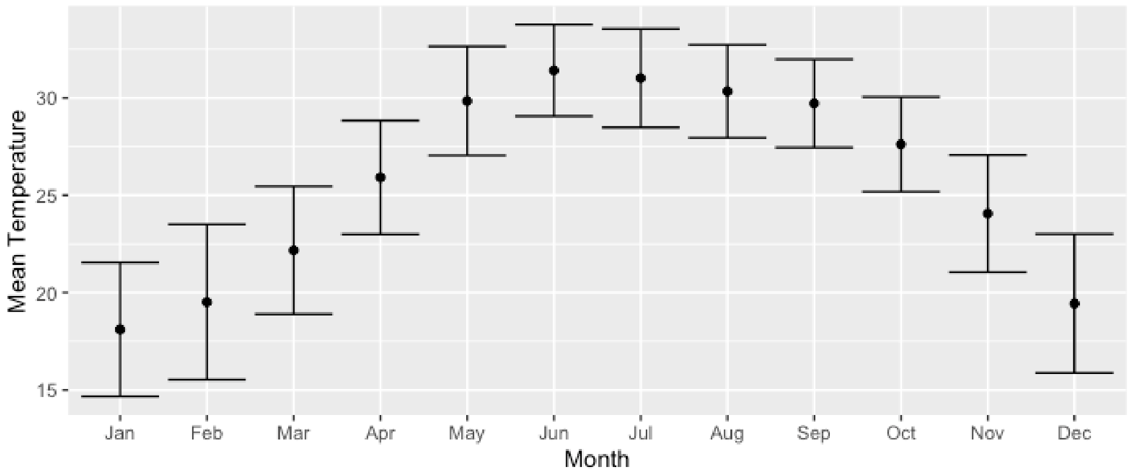

Figure S3: Monthly mean and standard deviations of daily mean temperature in Hanoi 2013-2019

Table S3: Pair-wise daily correlation coefficients between daily minimum, mean and maximum temperatures, daily relative humidity, and daily $PM_{2.5}$ in urban Hanoi, Vietnam, 2013-2019

Minimum temperature	1				
Mean temperature	0.96	1			
Maximum temperature	0.89	0.96	1		
Relative humidity	-0.08	-0.18	-0.27	1	
$PM_{2.5}$	-0.40	-0.36	-0.30	-0.04	1

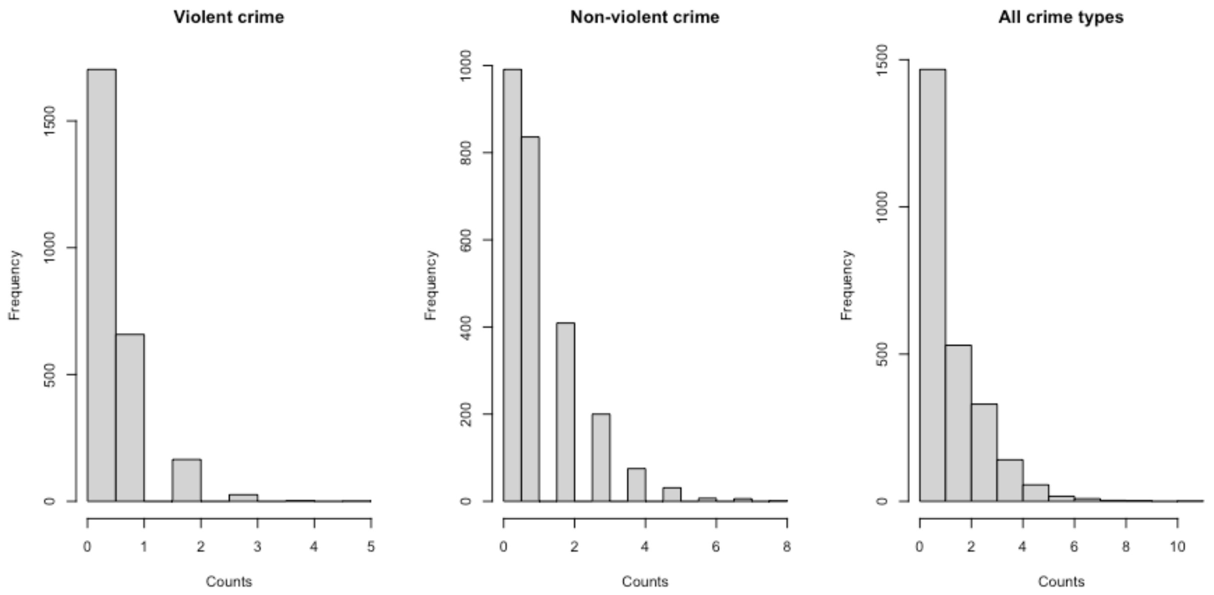

Figure S4: Distribution of daily crime counts in violent crime, non-violent crime, and total crime in Hanoi, Vietnam 2013-2019

Table S4: Akaike information criteria (AIC) values for linear effects models and non-linear effects models between temperature measures and crime

	**AIC**		
	**Violent crime**	**Non-violent crime**	**Total**
Linear regression models			
Minimum	4238	6869	7835
Mean	4236	6866	7830
Maximum	4235	6867	7831
Non-linear regression models			

Minimum	4125	6615	7561
Mean	4122	6613	7561
Maximum	4124	6630	7568

Model 1: Crude + public holiday + year

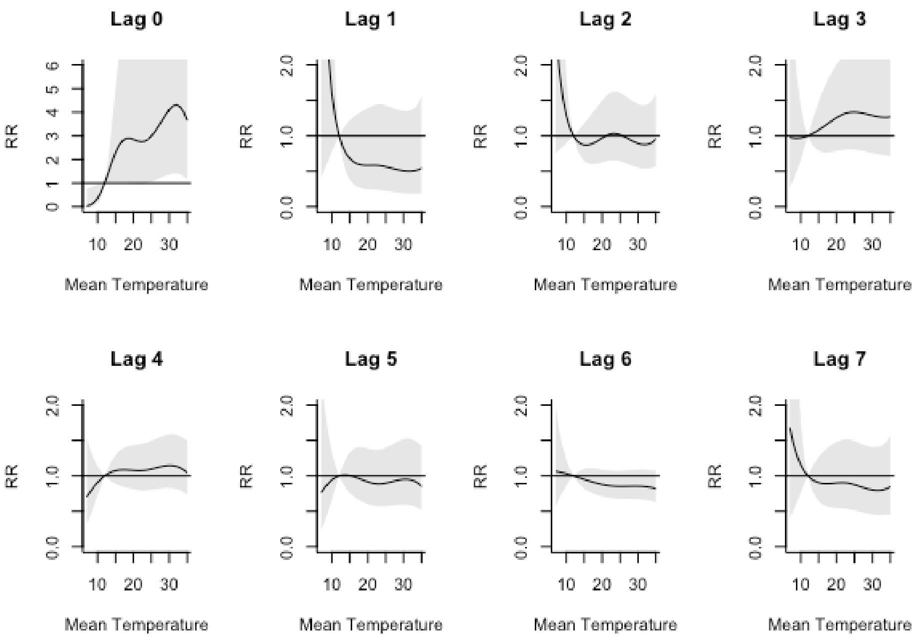

Figure S5: Lag-response curve at different lags day for violent crime in urban Hanoi, Vietnam, 2013-2019. Shaded areas denote 95% confidence intervals

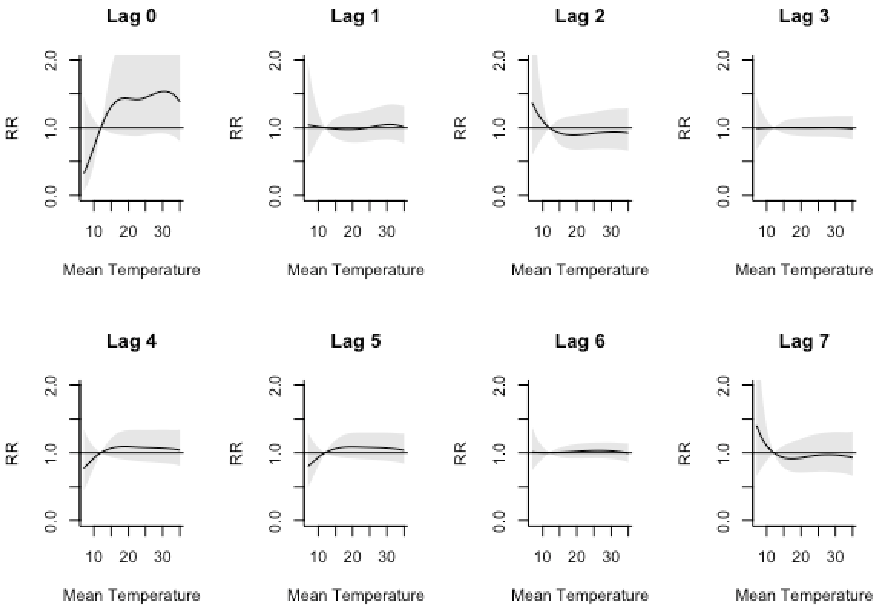

Figure S6: Lag-response curve at different lags day for non-violent crime in urban Hanoi, Vietnam, 2013-2019. Shaded areas denote 95% confidence intervals

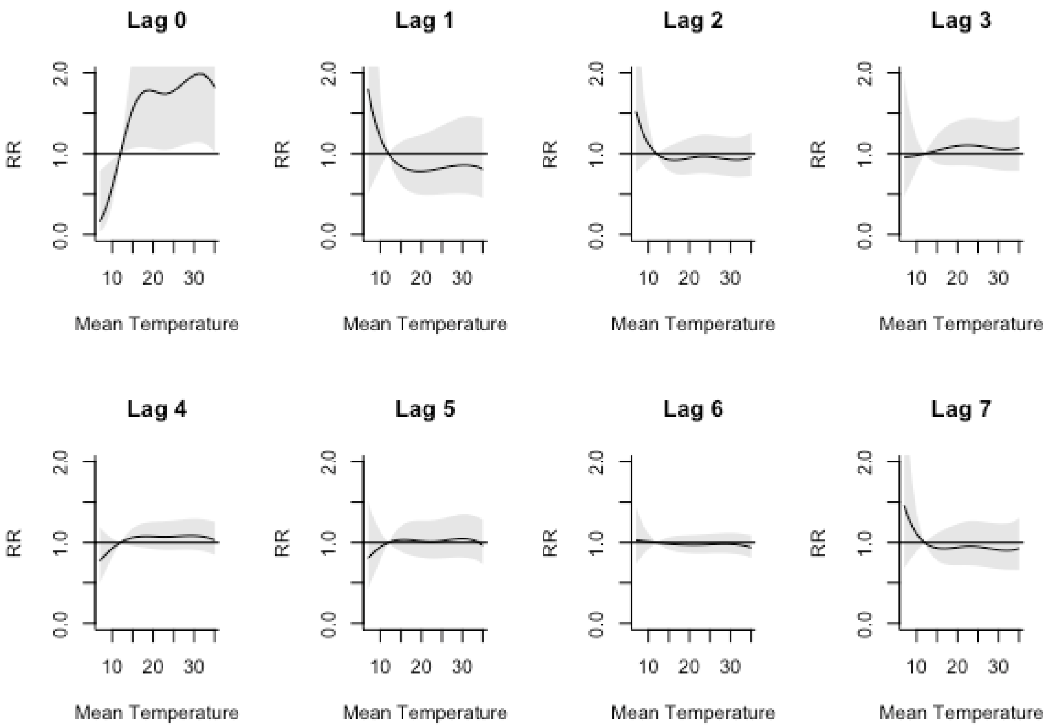

Figure S7: Lag-response curve at different lags day for all types of crime in urban Hanoi Vietnam, 2013-2019. Shaded areas denote 95% confidence intervals

Table S5: Sensitivity analysis of effect estimates for violent crime, non-violent crime and total crime using time series analysis by different degrees of freedoms for time

Degrees of freedom	**Violent crime**		**Non-violent crime**		**Total**	
	RRI1	95%CI	RRI1	95%CI	RRI1	95%CI
**3**	7.8	-4.5; 21.7	4.6	-0.2; 18.1	9.1*	2.8; 15.8
**5**	3.0	-9.7; 17.4	4.3	-1.4; 19.0	7.4*	0.7; 14.5
**7**	2.5	-10.2; 17.1	4.2	-2.4; 19.2	6.8*	0.1; 14.0
**9**	4.8	-8.2; 20	4.4	-1.5; 19.5	7.2*	0.5; 14.5

* p<0.05

Table S6: Mean and standard deviation of violent crime, non-violent crime and total crime by holidays and weekends

	**Number of days**	**Violent crime**		**Non-violent crime**		**Total crime**		**Mean temperature**	
	Total	Mean	SD	Mean	SD	Mean	SD	Mean	SD
**All days**	2556	0.42	0.68	1.1	1.22	1.52	1.42	25.84	5.56
**Non-holidays**	2417	0.43	0.68	1.11	NA	1.54	1.42	25.9	5.59
**Holidays**	137	0.33	0.60	0.85	NA	1.18	1.40	24.0	4.66
**Weekdays**	1826	0.43	0.68	1.12	NA	1.55	1.44	25.8	5.5
**Weekends**	730	0.41	0.67	1.04	NA	1.45	1.37	25.9	5.72

Table S7: Estimated relative risk of violent, non-violent, and total crime for each 5 °C increase in daily mean temperature with no lag (lag 0). Estimation from holidays and normal days, and weekends and weekdays

	**Violent crime**		**Non- Violent crime**		**Total crime**	
	RRI	95%PI	RRI	95%PI	RRI	95%PI
Non-holidays and Holidays						
Non- holiday	8.8	-0.9; 19.5	5.3	-0.9; 11.8	6.1*	0.9; 11.6
Holiday	123.5*	8.2; 361.5	95.9	29.3; 196.7	98.9*	38.2; 186.2
Weekdays and weekends						
Weekdays	10.2	- 1.1; 22.7	7.5*	0.3; 15.3	8.1*	1.9; 14.7
Weekends	7.5	-10.1 – 28.5	5.8	-5.6; 18.7	6.1	-3.4; 16.8

* $p<0.05$
